# Reuse of malaria rapid diagnostic tests for amplicon deep sequencing to estimate *Plasmodium falciparum* transmission intensity in western Uganda

**DOI:** 10.1038/s41598-018-28534-3

**Published:** 2018-07-05

**Authors:** Ross M. Boyce, Nick Hathaway, Travis Fulton, Raquel Reyes, Michael Matte, Moses Ntaro, Edgar Mulogo, Andreea Waltmann, Jeffrey A. Bailey, Mark J. Siedner, Jonathan J. Juliano

**Affiliations:** 1 Division of Infectious Diseases, University of North Carolina at Chapel Hill, 130 Mason Farm Road, Chapel Hill, 27599 USA; 2 Program in Bioinformatics and Integrative Biology, University of Massachusetts, 368 Plantation St., Worcester, Massachusetts 01605 USA; 3 Division of Epidemiology, University of North Carolina at Chapel Hill Gillings School of Global Public Health, 135 Dauer Drive, Chapel Hill, 27599 USA; 4 Division of General Medicine & Clinical Epidemiology, University of North Carolina at Chapel Hill, 5039 Old Clinic Building, CB 7110, Chapel Hill, 27599 USA; 5 Department of Community Health, Mbarara University of Science & Technology, P.O. Box 1410 Mbarara, Uganda; 6 Department of Medicine, Harvard Medical School and Massachusetts General Hospital, 125 Nashua Street, Suite 722, Boston, 02114 USA; 7 Curriculum in Genetics and Microbiology, University of North Carolina at Chapel Hill, 321 South Columbia Street, Chapel Hill, NC 27516 USA

## Abstract

Molecular techniques are not routinely employed for malaria surveillance, while cross-sectional, community-based parasite surveys require significant resources. Here, we describe a novel use of malaria rapid diagnostic tests (RDTs) collected at a single facility as source material for sequencing to esimtate malaria transmission intensity across a relatively large catchment area. We extracted *Plasmodium falciparum* DNA from RDTs, then amplified and sequenced a region of the apical membrane antigen 1 (*pfama1*) using targeted amplicon deep sequencing. We determined the multiplicity of infection (MOI) for each sample and examined associations with demographic, clinical, and spatial factors. We successfully genotyped 223 of 287 (77.7%) of the samples. We demonstrated an inverse relationship between the MOI and elevation with individuals presenting from the highest elevation villages harboring infections approximately half as complex as those from the lowest (MOI 1.85 vs. 3.51, AOR 0.25, 95% CI 0.09–0.65, *p* = 0.004). This study demonstrates the feasibility and validity of using routinely-collected RDTs for molecular surveillance of malaria and has real-world utility, especially as the cost of high-throughpout sequencing continues to decline.

## Introduction

To acheieve malaria elimination, there is a need for tools that accurately measure transmission intensity to identify target areas for public health interventions^1–3^. The multiplicity of infection (MOI), defined as the number of concurrent parasite clones per *Plasmodium falciparum*-infected host, has shown promise as a surrogate measure of malaria transmission intensity^4^. In the more than twenty years since its first descriptions^5–7^, the MOI has been employed in a variety of roles to include estimating malaria transmission intensity between differing areas^8,9^, assessing changes in transmission intensity over time^10,11^, and evaluating the impact of interventions ranging from chemoprophylaxis^12,13^ to vaccines^14,15^.

In malaria endemic areas, the presence of polyclonal infections is common, and caused by infection from multiple mosquitoes or infection from a single mosquito harboring multiple parasite clones^16^. The MOI is generally considered to be positively associated with the intensity of transmission. As shown in Table 1, significant differences in the MOI between sites of varying transmission intensity have been consistently demonstrated across sub-Saharan Africa^17–31^. Host factors such as premunition, however, also impact the MOI^5,7,32^. Efforts have been made to correlate the MOI with age, parasite density, and disease severity, all of which reflect host immunity, with mixed results (Table 1).

**Table 1:** Representative selection of previous studies from sub-Saharan Africa demonstrating the relatively consistent association between the multiplicity of infection (MOI) and malaria transmission intensity, contrasted with the more variable associations between MOI and patient age, parasite density, and malaria severity.

Study	Country	Population	Markers	Transmission	Age	Parasitemia	Severity
A-Elbasit *et al*. (2007)	Sudan	Symptomatic	*msp-2*	—	No	No	No
Amodu *et al*. (2008)	Nigeria	Symptomatic	*msp-2*	—	—	—	Yes*
Apinjoh *et al*. (2015)	Cameroon	Symptomatic	*msp-1*	—	No	No	—
Bendixen *et al*. (2001)	Tanzania	Asymptomatic	*msp-1/msp-2*	**Yes**	**Yes**	**Yes**	—
Durand *et al*. (2008)	Madagascar	Symptomatic	*msp-2*	**Yes**	No	—	No
Engelbrecht *et al*. (2000)	Nigeria	Asymptomatic	*msp-2*	—	**Yes**	—	—
Francis *et al*. (2006)	Uganda	Symptomatic	*msp-2*	**Yes**	—	—	—
Kateera *et al*. (2016)	Rwanda	Symptomatic	*msp-2*	**Yes**	**Yes**	**Yes**	—
Kiwuwa *et al*. (2013)	Uganda	Symptomatic	*msp-1/msp-2/csp/glurp*	—	No	—	**Yes** ^**^
Mahdi *et al*. (2016)	Sudan	Symptomatic	*msp-1/msp-2*	—	No	—	No
Manjurano *et al*. (2011)	Tanzania	Asymptomatic	*msp-2*	**Yes**	No	—	—
Mayor *et al*. (2007)	Mozambique	Asymptomatic	*msp-2*	—	**Yes**	**Yes**	—
Mockenhaupt *et al*. (2003)	Ghana	Asymptomatic	*msp-2*	—	No	No	**Yes** ^**#**^
Peyerl-Hoffmann *et al*. (2001)	Uganda	Asymptomatic	*msp-1/msp-2*	No	**Yes**	**Yes**	—
Shigidi *et al*. (2004)	Sudan	Mixed	*msp-2*	—	—	—	**Yes** ^**##**^

^*^Lower MOI associated with severe malaria.

^**^Higher MOI associated with severe malaria.

^#^*Higher MOI associated with severe malaria anemia*.

^##^*Higher MOI associated with cerebral malaria*.

To estimate MOI, investigators have historically collected dried blood spots (DBS), which allow long-term storage of malarial DNA. However, DBS are not collected in routine clinical practice, and require additional supplies for sampling and storage, which generally precludes estimation of MOI outside of research studies. Moroever, the MOI is traditionally determined using nested PCR (nPCR) with gel electrophoresis to detect polymorphisms in the highly variable surface antigens of the merozoite surface proteins (*msp-1* & -2) or the glutamate-rich protein (*glurp*)^33^. However, these techniques are labor intensive and often fail to detect all sequence polymorphisms and low-abundance variants^34,35^. Newer methods utilizing targeted amplicon deep sequencing of malaria infections provide a high-throughput, highly sensitive approach for detecting clones in polyclonal infections as well as more accurate quantitative estimates of clonal frequency^36^.

In this study, we investigated the use of a targeted amplicon deep sequencing approach to describe the clonal diversity of the *P. falciparum* apical membrane antigen 1 (*pfama1*) across a geographically diverse area of western Uganda. To achieve this, we extracted DNA from malaria rapid diagnostic tests (RDTs) collected during routine care at a single rural health facility and stored at room temperature under tropical conditions. Our primary objective was to identify spatial differences in the MOI as a surrogate marker of malaria transmission intensity over a highland area using only a facility-based sample of routinely-collected rapid tests. While RDTs have previously been used as a source of malarial DNA for PCR and the sequencing of drug resistance and mitochondrial polymorphisms, they have not been employed to estimate transmission intensity^37–41^. Our relatively efficient sampling strategy precludes the need for more resource-intensive, population-based approaches to mapping transmission intensity and parasite diversity. Secondary objectives included an investigation of associations between the MOI, age, and the clinical spectrum of malaria, as well as an exploration of the spatial micro-epidemiology of parasites based on the *pfama1* haplotypes.

## Materials and Methods

### Study setting

**Figure 1:**
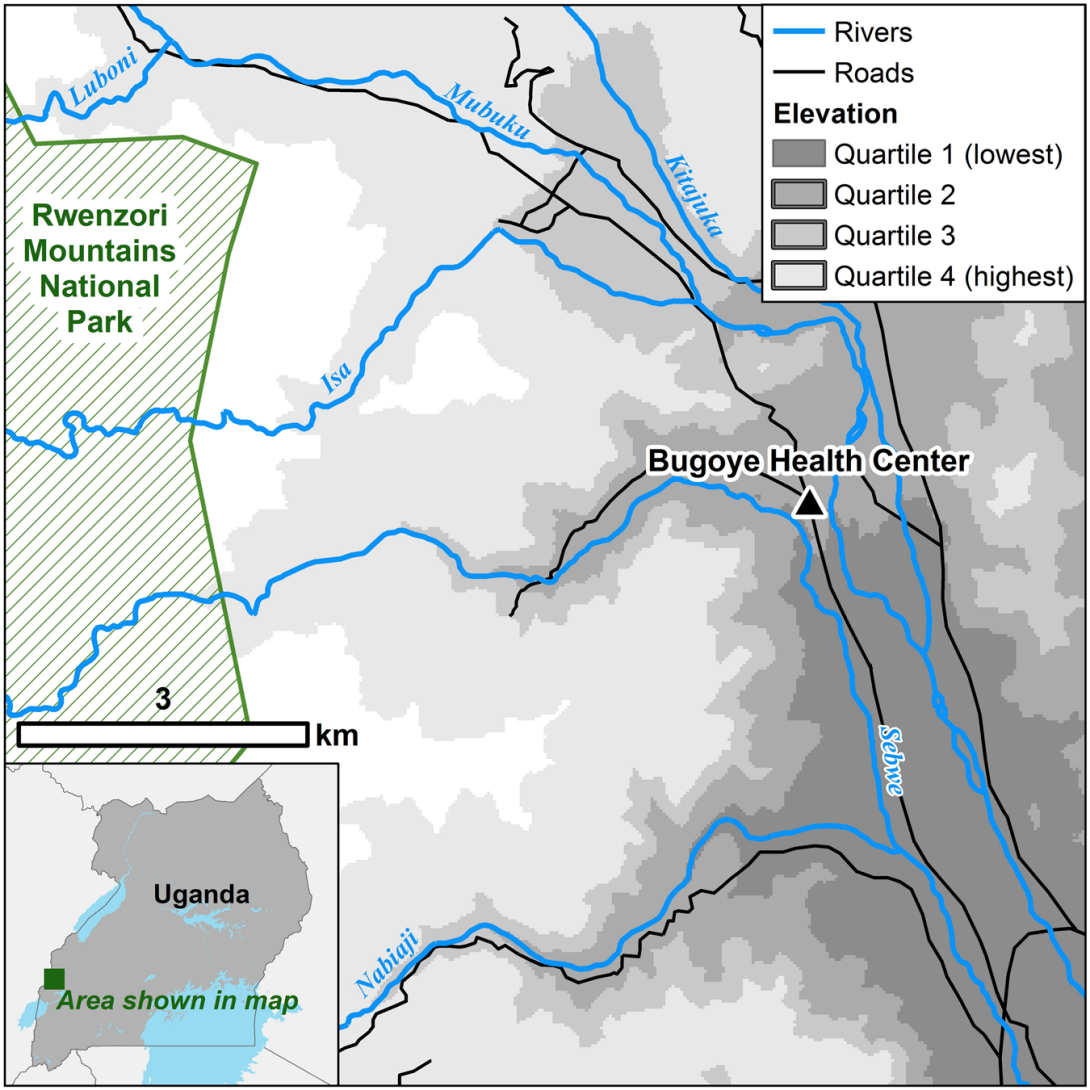
Map of the study area shaded by elevation quartiles. Map created using ArcGIS, Version 10.4.1 (ESRI, Redlands, CA) available at http://desktop.arcgis.com/en/.

Samples were collected from patients presenting with febrile illness to the Bugoye Level III Health Center in the Kasese District of western Uganda. The geography of the study area is highly varied. The westernmost villages of the sub-county are characterized by deep river valleys and steep hillsides with elevations up to 2,000 meters. In contrast, villages located to the east are defined by low-lying, level terrain (Fig. 1). Three large rivers (the Mubuku to the north, Sebwe bisecting the sub-county, and the Nambiaji to the south) flow down valleys from west to east converging in the low-lying basin area near the health center. Like much of Uganda, the climate in Bugoye permits year-round malaria transmission marked by semi-annual transmission peaks typically following the end of the rainy seasons^42^. The two most recent malaria indicator surveys undertaken in the region found declining parasitemia prevalence from 48.4% to 17.6% in 2009 and 2014, respectively^43,44^.

### Sample Selection

Rapid diagnostic tests were performed as part of the Rapid Diagnostic Tests for Severe Malaria (RDTSM) study, a prospective, observational cohort study of patients with a parasitological diagnosis of malaria conducted from May to November 2015. We have reported full details of the study methodology elsewhere^45^.

In brief, initial testing for malaria was performed using the Standard Diagnostics 05FK60 Malaria Ag *P.f*/Pan RDT assay (Standard Diagnostics, Hagal-Dong, Korea). Study RDT were obtained directly from the manufacturer, stored in the original packaging at room temperature, and utilized in accordance with the manufacturer’s instructions prior to the expiry date. Each day, study staff packaged completed RDTs in a polyethylene bag with desiccant. RDTs were stored at room temperature for approximately two years prior to extraction. Study staff with training in laboratory medicine prepared thin and thick blood smears for all patients with a positive RDT result. Hemoglobin levels were measured using the Hemocue Hb 201+ analyzer (Brea, CA), and venous lactate values were obtained using the Abbott iStat analyzer (Princeton, NJ).

We first stratified the RDT-positive results by village. From each village-based strata, we sorted by level of parasitemia as determined by microscopy and systematically sampled starting from the highest density sample to achieve a balance of high and low parasitemias within each village-based strata. A convenience sample of approximately sixteen RDTs from each village was selected. *Post hoc*, we found that this number was more than sufficient to power the primary analysis at a level of 0.80 (α = 0.05) to detect the observed mean difference in MOI between the highest and lowest quartiles of elevation, even accounting for failed genotyping in nearly a quarter of samples.

### Laboratory and Bioinformatic Methods

We removed a 1 cm section of the internal filter paper from the RDTs according to a previously described protocol (Supplementary Fig. S1)^40^. DNA was extracted using a previously described Chelex extraction method in a final volume of 100 μL^46^. The concentration of extracted *P. falciparum* DNA in individual samples was determined using a quantitative real-time PCR (qPCR) for *P. falciparum* lactate dehydrogenase (*pfldh*)^47^. *Pfama1* was amplified from individual samples in duplicate in a hemi-nested PCR modified from Miller *et al*., which was shown to reproducibly and accurately determine the frequency and haplotype of variants in known mixtures in our laboratory using Ion Torrent sequencing^48^. In this approach, the inner forward primers are labeled with a unique barcode (Supplementary Table S1), referred to as an MID, which allows for multiple PCR products to be pooled prior to sequencing library preparation (Supplemental Fig. S2)^48^. We employed 22 unique MIDs, allowing 11 samples to be sequenced in duplicate in each Illumina library. Each PCR within a final sequencing library contained a unique MID, thus allowing the PCR replicate for each clinical sample to be de-convoluted bioinformatically as described below.

Modifications to the PCR protocol included using 2.5 units of Roche FastStart high fidelity *Taq* polymerase, and final concentrations of 1.8 mM for MgCl_2_, 200 uM for each dNTP (dATP, dCTP, dGTP, dTTP), and 167 nM for forward and reverse primers for each step of the hemi-nested PCR. Thus, all samples were amplified and uniquely barcoded in duplicate. Successful amplification was confirmed by visualizing the qPCR products on a 1% agarose gel and the product was quantified using Quantifluor dsDNA System (Promega, Fitchburg, WI) on a Synergy HT Multi-Mode Microplate Reader (BioTek, Winooski, VT). Barcoded samples were then pooled in equal amounts based on concentration. Pools of MID labeled PCR products were cleaned using KAPA Pure Beads (Illumina, San Diego, CA) and multiple indexed libraries were prepared for sequencing using KAPA Hyper Library Prep kits (Illumina, San Diego, CA) using NEXTflex DNA barcodes (BiooScientific, Austin, TX). The indexed pools were then combined in equimolar concentration into a final pool that was sequenced on a part of a MiSeq (2 × 300 bp chemistry) at the University of North Carolina High Throughput Sequencing Facility. Unlike Ion Torrent, Illumina sequencers have problems handling samples with low heterogeneity. We typically use two approaches to increase thje heterogeneity on the sequencing runs. First, we include 10% PhiX spike in all of our amplicon deep sequencing runs. Second, we often run libraries for other projects on the same run as the amount of sequencing necessary for a small project is not sufficient to warrant a complete run. This library comprised an estimated 80% of the run which also contained amplicons from other studies.

For these experiements, we included a positive control comprised of a known mixture of three malaria strains: 3D7 (MRA-102G, BEI Resources, Manansas, VA), FCR3 (MRA-321G, BEI Resources), and Dd2 (MRA-150G, BEI Resources). Using this control mixture, *pfama1* amplicons were generated for 5 controls, representing 10 MID labeled PCR reactions, and sequenced alongside the clinical samples. To ensure that we correctly estimated the frequency of the three strains in the mixture, we amplified the mixture using the same conditions using only the second round reaction for 30 cycles. We cloned the PCR product using a TOPO TA cloning kit (Invitrogen) and performed Sanger sequencing of 22 colonies using the M13F primer at GENWIZ (RTP, NC). Sequences were analyzed using Geneious R10 (Biomatters Inc., Newark, NJ). We estimated allele frequencies and confidence intervals by the bootsrap method with 1,000 replicate bootstraps. This was compared to the allele frequency determined by the four replicate control deep sequencing reactions.

The deep sequencing reads were de-multiplexed and clustered using the software package, SeekDeep (http://baileylab.umassmed.edu/SeekDeep), as previously described^49^. This approach uses the PCR replicates for each sample to reduce PCR and sequencing errors as shown in the SeekDeep Process Cluster step of Fig. 1 of Hathaway *et al*., and has been shown to provide accurate frequency and genotype determinations with as few as 200 reads per sample^49^. As haplotypes in each PCR are called independently and then only haplotypes conserved between replicate PCRs are used, this provides a conservative approach for haplotype detection to determine MOI. Here, samples were included in the final analysis if they had ≥250 total reads combined between the replicates. The low read coverage in some samples did not influence the MOI estimates as seen in Supplemental Fig. S5. Haplotypes were included in the analysis if ≥80% of haplotype reads had a Phred Quality Score of ≥30 and if they occurred in both sequencing replicates and were above an averaged frequency cutoff of 2.5% between the two PCRs, similar to our previous work (i.e. if the within-sample average frequency across duplicate runs was above this cutoff)^48^. Haplotypes that were marked as likely chimeric were excluded. Haplotypes in each sample were compared to all identified haplotypes in order to provide population-level statistics. All data generated or analyzed during this study are included in this published article and the Sequence Read Archive available at www.ncbi.nlm.nih.gov/sra (SRA Accession Number Pending).

### Statistical analysis

We first summarized demographic, clinical, and laboratory characteristics of the cohort using Student’s t-test for continuous variables and Pearson’s Chi squared test for categorical variables. We defined severe malaria in accordance with the WHO guidelines for research and epidemiological studies using a threshold of ≥250,000 parasites/μl to define hyperparasitemia^50^.

The MOI was calculated as the number of concurrent parasite clones per *P. falciparum*-positive sample. We performed ordinal logistic regression to explore the demographic, spatial, clinical and laboratory parameters associated with the MOI, the primary outcome measure of interest. Age categories were set to be comparable with prior studies that found significant associations between age and MOI^22,30^. All variables that were significant in univariate models with a pre-specified *P*-value of <0.25 were included in the subsequent multivariate analysis^51^. We compared the results of the regression analysis to the RDT positivity rate, defined as the number of positive malaria tests per 100 suspected cases examined.

To assess the potential relationship between the MOI and disease severity, we selected three outcomes of interest: (a) lactic acid levels, (b) hemoglobin levels, and (c) severe malaria. For the continuous outcomes of lactic acid and hemoglobin levels, we first performed linear regression with MOI serving as the primary explanatory variable. We then utilized negative binomial general linear regression model with a log-link function and robust standard errors to explore associations between the categorical outcomes of (a) lactic acidosis (venous lactate ≥5 mmol/L), (b) anemia (hemoglobin <7 g/dL), and (c) severe malaria. Data were analyzed with Stata 12.1 (College Station, TX).

To estimate associations between geographic factors and the MOI, we completed village-level geographic information system (GIS) mapping of the sub-county and surrounding environs, comprising an area of approximately 55 square kilometers. These data were entered into ArcGIS, Version 10.4.1 (ESRI, Redlands, CA) to create a reference map, from which we calculated the mean elevation and area of each village and subsequently divided the data into quartiles of elevation.

### Ethics statement

Ethical approval of the study was provided by the institutional review boards of the University of North Carolina at Chapel Hill, the Mbarara University of Science and Technology, and the Uganda National Council for Science and Technology. Written informed consent was obtained from all adult study participants and the caregivers of participating children. All research was performed in accordance with relevant guidelines and regulations.

## Results

### Validation of control mixtures

Allele frequency calls for all five control mixtures are shown in Supplemental Fig. S3. Using a previously described measure of concordance for allele frequency calls between PCR replicates (*d*_o_), the replicate PCRs showed a high level of agreement of allele frequencies in each of the five controls with a mean *d*_o_ of 0.02^52^. Using data from the cloned Sanger sequences as the reference standard, we evaluated the accuracy of the deep sequencing frequency estimates. We bootstrapped the frequency estimates to generate confidence intervals for the Sanger sequence data and compared it to the variation in replicate deep sequencing reactions (Supplementary Fig. S4). The amplicon deep sequencing method provided accurate estimates of haplotype frequency, suggesting that haplotype frequencies in the clinical samples were representative. No false haplotypes were detected in the control mixtures.

### Extraction of genomic DNA from RDTs and amplification of samples

We detected *pfldh* malarial DNA and were subsequently able to amplify *pfama1* in 287 of 299 (96.0%) of RDT positive samples. Of the twelve RDTs from which we were unable to detect *pfldh or pfama1*, four were negative on microscopy while another four had parasite densities of <250 parasites/μl. We successfully sequenced 223 of the 287 (77.7%) samples with detectable malarial DNA. Demographic and clinical characteristics of the cohort are summarized in Table 2. There were no significant differences in age (*p* = 0.28), sex (*p* = 0.85), parasitemia (*p* = 0.29), or disease severity (*p* = 0.70) between those samples successfully genotyped and those that were excluded from the analysis.

**Table 2:** Baseline demographic, laboratory, and clinical characteristics of cohort.

	Total	Gentoyped	Excluded	*p*-value
Patients (n, %)	287 (100)	223 (77.7)	64 (22.3)	—
Age (median, IQR)	12 (7–20)	12 (7–20)	12 (6–22.5)	0.28
Female	144 (50.5)	111 (50.2)	33 (51.6)	0.85
Febrile	71 (25.8)	60 (28.3)	11 (17.5)	0.08
**Microscopy**				
*P. falciparum*	267 (95.7)	211 (97.7)	56 (88.9)	**0.008**
Mixed Infection	6 (2.2)	3 (1.4)	3 (4.8)	
Negative	6 (2.2)	2 (0.9)	4 (6.4)	
Parasite Density (GM, IQR)	9,701/μl (3,318–28,001)	10,400/μl (3,623–26,488)	8,531/μl (2,058–39,838)	0.29
<2,500/μl	61 (21.3)	45 (20.2)	16 (25.0)	0.59
2,500–9,999/μl	78 (27.2)	60 (26.9)	18 (28.1)	
10,000–99,999/μl	105 (36.6)	86 (38.6)	19 (29.7)	
≥100,000/μl	43 (15.0)	32 (14.4)	11 (17.2)	
Lactate (mean, 95% CI)	2.02 (1.89–2.15)	2.00 (1.87–2.14)	2.07 (1.72–2.43)	0.66
Hemoglobin (mean, 95% CI)	12.2 (11.9–12.5)	12.2 (11.9–12.5)	12.3 (11.8–12.9)	0.78
Severe Malaria	28 (10.7)	21 (10.3)	7 (12.1)	0.70

### Multiplicity of Infection

After filtering in SeekDeep, we used 5.56 million of 8.15 million sequencing reads to call AMA haplotypes and determine multiplicity of infection in each sample. The mean number of reads used per sample was 22,763 (range 254–120,213). Read depth for each sample stratified by MOI is shown in Supplementary Fig. S5. The mean and median MOI was 3.09 (95% CI 2.74–3.44) and 2.0 (IQR 1.0–4.0), respectively. The crude monthly mean MOI peaked in May (MOI = 4.68, 95% CI 3.22–6.14), which is the last month of the traditional rainy season, and then declined significantly, reaching a nadir in October, (MOI = 2.06, 95% CI 1.06–3.07, *p* = 0.003 compared to May), which marks the beginning of the second rainy season.

**Figure 2:**
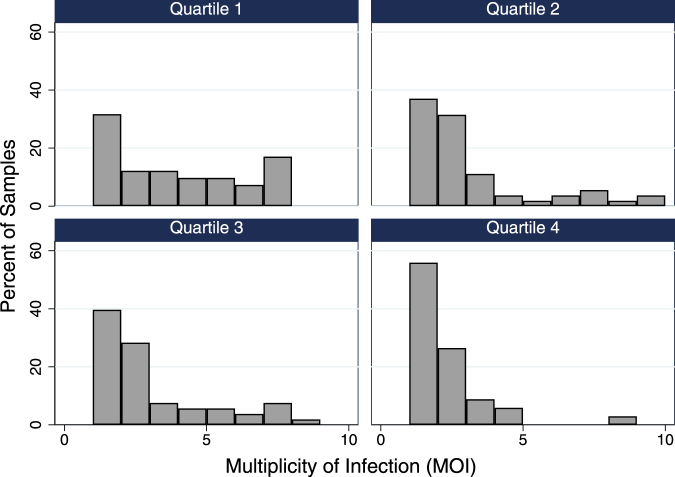
Multiplicity of infection (MOI) stratified by elevation quartiles showing that mono-infections comprised the smallest proportion of infections in the lowest elevation villages (Quartile 1) and the highest proportion in the highest villages (Quartile 4).

The MOI varied significantly with geographic factors, including village elevation and river valley of residence. The MOI demonstrated an inverse relationship with elevation (Table 3). On average, individuals presenting from the highest elevation villages harbored infections approximately half as complex as those from the lowest elevation villages (MOI 1.85 vs 3.51, OR 0.24, 95% CI 0.10–0.57, *p* = 0.001) (Fig. 2). This finding was robust in the final model, which was adjusted for age and disease severity. We also observed a significantly lower MOI among individuals presenting from the Sebwe (MOI 2.28, aOR 0.55, 95% CI 0.31–0.97, *p* = 0.04) and Nambiaji (MOI 1.75, aOR 0.31, 95% CI 0.11–0.85, *p* = 0.02) river valleys compared to the basin area (MOI 3.25) where the rivers converge. These results were generally consistent with the RDT positivity rates observed in each valley with both the Sebwe (aOR 0.84, 95% CI 0.72 – 0.0.97, *p* = 0.02) and Nambiaji (aOR 0.64, 95% CI 0.50–0.81, *p* < 0.001) river valleys being significantly lower than the basin area.

**Table 3:** Mean multiplicity of infection (MOI) by sub-group and ordinal logistic regression modeling of correlates of MOI.

Variable	Mean MOI	OR (95% CI)	*p-*Value	aOR (95% CI)	*p-*Value
**Sex**					
Female	3.16 (2.65–3.67)	REF	REF	—	—
Male	3.00 (2.51–3.49)	0.89 (0.56–1.44)	0.64	—	—
**Age Category**					
<3 years	2.16 (1.09–3.23)	REF	REF	REF	REF
3–4 years	3.47 (1.99–4.96)	2.82 (0.81–9.86)	0.10	1.11 (0.19–6.60)	0.91
5–7 years	3.00 (1.80–4.20)	2.37 (0.72–7.82)	0.16	1.57 (0.26–9.41)	0.63
8–11 years	2.97 (2.21–3.74)	2.50 (0.92–6.84)	0.07	1.45 (0.33–6.39)	0.62
12–17 years	3.05 (2.44–3.66)	2.51 (0.97–6.52)	0.06	1.35 (0.32–5.66)	0.68
18–29 years	3.46 (2.60–4.33)	3.17 (1.14–8.78)	**0.03**	1.53 (0.36–6.52)	0.57
≥30 years	3.26 (1.98–4.54)	2.39 (0.74–7.75)	0.15	0.85 (0.16–4.)	0.84
**Parasitemia**					
<2,500/μl	3.51 (2.55–4.47)	REF	REF	—	—
2,500–9,999/μl	3.52 (2.75–4.29)	1.22 (0.53–2.84)	0.64	—	—
10,000–99,000/μl	2.80 (2.34–3.27)	0.96 (0.45–2.06)	0.91	—	—
≥100,000/μl	2.47 (1.79–3.15)	0.77 (0.32–1.85)	0.56	—	—
**Severity**					
Uncomplicated	3.25 (2.86–3.64)	REF	REF	REF	REF
Severe	2.29 (1.46–3.12)	0.56 (0.27–1.16)	0.12	0.60 (0.23–1.52)	0.28
Elevation*					
Quartile 1	3.51 (2.77–4.26)	REF	REF	REF	REF
Quartile 2	2.70 (2.08–3.33)	0.51 (0.23–1.12)	0.09	0.42 (0.17–1.02)	0.06
Quartile 3	2.62 (2.06–3.19)	0.49 (0.22–1.08)	0.08	0.45 (0.20–1.04)	0.06
Quartile 4	1.85 (1.33–2.37)	0.24 (0.10–0.57)	**0.001**	0.25 (0.09–0.65)	**0.004**

Elevation ranges: Quartile 1 = 1136–1225 m, Quartile 2 = 1259–1339 m, Quartile 3 = 1355–1424 m, Quartile 4 = 1451–1830 m

Abbreviations: OR = odds ratio, aOR = adjusted odds ratio.

The highest MOI was found in children age 3 to 4 years of age (MOI = 3.47, 95% CI 1.99–4.96) compared to those aged 8–11 years of age, who had the lowest MOI (2.97, 95% CI 2.21–3.74). Differences by age, however, were not significant as shown in Table 3 or when age groups were broadened to include children <5 years of age, 5 to 15 years of age, and ≥15 years of age (Supplementary Table S2). No clinical factors were associated with the degree of infection complexity. While there were trends towards lower MOI with severe disease in the univariate regression analysis, these findings were not significant in adjusted models.

When we defined severe malaria as the outcome measure of interest, we found strong correlations with male sex, age less than five years, and parasite density greater than 100,000 parasites/μl in the 21 cases of severe disease (Table 4). There was, however, no significant difference in the proportion of patients with polyclonal infections among patients with uncomplicated versus severe malaria (66.1 vs. 57.1%, *p* = 0.41). Increasingly complex infections (≥3 identified haplotypes) demonstrated a trend towards a reduced risk of severe malaria although this finding was not significant in the multivariate model (aIRR 0.65, 95% CI 0.25–1.73, *p* = 0.39), which was adjusted for age. Of note, when the analysis was stratified by age, we found that risk of severe malaria was non-significantly higher with more complex infections (IRR = 1.67, 95% CI 0.34–8.06, p = 0.53) in children <5 years of age, while the risk of severe malaria trended lower with more complex infections in children and adults ≥5 years of age (IRR 0.24, 95% CI 0.05–1.12, *p* = 0.07).

**Table 4:** Negative binomial regression modeling of correlates of severe malaria (n = 21).

Variable	Risk Ratio (IRR)	95% CI	*p-*Value	Adjusted RR	95% CI	*p-*Value
**Sex**						
Male	1.86	0.89–3.87	0.10	3.05	1.40–6.64	**0.005**
**Age Category**						
≥15 years	REF	REF	REF	REF	REF	REF
5 to 14 years	2.62	0.99–6.93	**0.05**	5.90	0.77–45.2	0.09
<5 years	4.70	1.60–13.8	**0.005**	8.39	1.06–66.6	**0.04**
**Parasitemia**						
<2,500/μl	REF	REF	REF	REF	REF	REF
2,500–10,000/μl	0.76	0.11–5.25	0.78	0.42	0.04–4.76	0.48
10,000–100,000/μl	3.49	0.81–15.1	0.09	1.12	0.28–4.46	0.88
≥100,000/μl	10.7	2.54–44.9	**0.001**	5.38	1.39–20.8	**0.02**
**Fever**						
Febrile	2.24	1.09–4.56	**0.03**	1.14	0.53–2.49	0.73
**MOI Category**						
MOI=1	REF	REF	REF	REF	REF	REF
MOI=2	1.06	0.42–2.67	0.91	1.47	0.57–3.78	0.42
MOI≥3	0.49	0.17–1.39	0.18	0.65	0.25–1.73	0.39

Similarly, there was no association between the MOI and disease severity when we defined lactic acidosis (>5 mmol/L) or anemia (Hb < 7 g/dL) as the outcome measure of interest. While lactic acidosis and anemia were relatively rare outcomes in the cohort, we did not observe any trend between lactic acid (β coefficient = 0.02, 95% CI −0.03–0.07, *p* = 0.40) or hemoglobin levels (β coefficient = 0.07, 95% CI −0.04–0.17 *p* = 0.20) and the MOI in the linear regression models.

### Haplotype Analysis

A total of 39 unique haplotypes were identified [SRA Accession Number Pending]. The most common haplotype (UgandaMOI.00) was found in 122 of 223 (54.7%) of the included samples and accounted for 24.6% of the population fraction. The distribution of haplotypes in the population are shown in Supplementary Fig. S6. Approximately two thirds of included samples (n = 139, 62.3%) demonstrated polyclonal infections with the highest proportion observed in individuals 18 to 30 years of age (n = 28, 68.3%). In contrast, monoclonal infections were most common in children <3 years of age (n = 11, 57.9%).

**Figure 3:**
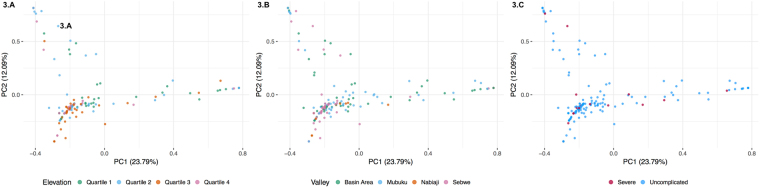
Principle component analyses (PCA) depicting the absence of population structure between individual haplotypes and quartiles of elevation (3.**A**), river valleys (3.**B**), or disease severity (3.**C**).

In the clustering analysis, we did not identify any evidence of population structure based on either haplotype prevalence (the proportion of infections containing the respective haplotype), or haplotype relative abundance (the proportion of the respective haplotype within an individual infection), when plotting by elevation, river valley or the presence/absence of severe malaria. (Fig. 3).

## Discussion

We carried out targeted amplicon deep sequencing from DNA extracted from routinely-collected RDTs among a facility-based cohort of symptomatic individuals and were able to demonstrate a significant, inverse correlation between village elevation and the MOI, consistent with previous observations^27^. We also found a lower MOI in two of the river valleys, which correlated with the RDT positivity rate, a crude measure of transmission intensity. Our findings are proof-of-concept that it is possible to estimate transmission intensity across a wide catchment area using only a facility-based sample of routinely-collected RDTs, thus negating the need for additional DBS collection or labor-intensive cross-sectional sampling. Such an approach has relatively broad programmatic application and may even be cost-effective when considering the labor costs associated with large cross-sectional surveys.

In contrast, we found no evidence of population structure by elevation or river valley based on the *pfama1* haplotypes. This outcome is not entirely unexpected, as it likely reflects the high intensity of transmission and resulting mixing of parasite sub-populations across the study site. It is also supported by our recent work showing no population structure based on this gene between distant geographic sites in Africa^48^. We had hypothesized that the high ridgelines separating the three major rivers could serve as a physical barrier to vector migration, isolating each valley from the next. Our results, however, could suggest that this barrier is inadequate to maintain population structure, most likely at the lower elevation areas where the rivers merge into the basin area. In addition, human movement into lower elevation areas, where most commercial activity occurs, could also facilitate the mixing of parasite populations between river valleys. Alternatively, the lack of structure could also result from the choice of marker. Antigens like *pfama1* may be under convergent evolution and thus similar patterns of diversity emerge in distant populations, as supported by our previous work^53,54^.

We did not identify any associations between the MOI and age or the MOI and markers of disease severity (i.e. anemia, lactate, severe malaria). However, given the few cases of severe malaria (n = 21), our analysis was only powered to a level of 0.54 to detect a mean difference of one parasite clone between cases of uncomplicated and severe malaria. Interestingly, we did observe a divergent effect of infection complexity on the risk of severe malaria among children <5 years of age, who had a relatively negligible risk of severe disease with a more complex infection (≥3 identified haplotypes), compared to adults and children ≥5 years of age, who had a clear trend (*p* = 0.07) towards a reduced risk of severe disease with more complex infections. This finding supports the hypothesis of age-dependent immune response mechanisms and is consistent with a report from Tanzania in which children <3 years of age experienced a greater risk of a subsequent malarial episode with increasing infection complexity, whereas in older children, more complex infections were associated with a decreased risk of clinical malaria,^55^.

Our study has several limitations. First, we did not utilize traditional measures of transmission intensity such as the entomological inoculation rate (EIR) or parasite prevalence against which we could compare our results. However, the relationship between elevation and transmission intensity is well established^56^ and thus we are confident that our findings in regard to the association between the MOI and elevation are valid. Second, we did not perform genotyping from samples stored on DBS, which would have allowed direct comparison between the two methods of sample collection, although previous studies have reported similar PCR success rates with each approach^38,40^. Lastly, we were unable to genotype 64 (22.3%) of our samples. This is likely a result of our conservative duplicate reading thresholds. We are reassured that there were no significant differences in demographic, clinical, or laboratory parameters between those samples that were successfully genotyped and those that were not.

## Conclusions

Using routinely-collected malaria RDTs from a single health facility as source material, we were able to deep sequence and estimate malaria transmission intensity across a large and geographically diverse catchment area. To our knowledge, this is the first study to demonstrate the feasibility and validity of such an approach, which we believe has practical implications for malaria surveillance programs, especially as the cost of high-throughput sequencing continues to decline. Similar techniques may also be applicable to other disease conditions where lateral flow assays are commonly utilized for point-of-care diagnosis.

## Electronic supplementary material

Supplementary Information 
