# Supplementary material for: Reuse of malaria rapid diagnostic tests for amplicon deep sequencing to estimate Plasmodium falciparum transmission intensity in western Uganda
Source: Sci Rep. 2018 Jul 5;8:10159. doi: 10.1038/s41598-018-28534-3 (PMC6033881; doi:10.1038/s41598-018-28534-3)

Reuse of malaria rapid diagnostic tests for amplicon deep sequencing to estimate *Plasmodium falciparum* transmission intensity in Western Uganda.

Ross M. Boyce[1*], Nick Hathaway[2], Travis Fulton[3], Raquel Reyes[4], Michael Matte[5], Moses Ntaro[5], Edgar Mulogo[5], Andreea Waltmann[1], Jeffrey A, Bailey[2], Mark J. Siedner[6], and Jonathan J. Juliano[1,3,7]

[1]Division of Infectious Diseases, University of North Carolina at Chapel Hill, 130 Mason Farm Road, Chapel Hill, 27599, USA

[2]Program in Bioinformatics and Integrative Biology, University of Massachusetts, 368 Plantation St., Worcester, Massachusetts 01605, USA

[3]Division of Epidemiology, University of North Carolina at Chapel Hill Gillings School of Global Public Health, 135 Dauer Drive, Chapel Hill, 27599, USA

[4]Division of General Medicine & Clinical Epidemiology, University of North Carolina at Chapel Hill, 5039 Old Clinic Building, CB 7110, Chapel Hill, 27599, USA

[5]Department of Community Health, Mbarara University of Science & Technology, P.O. Box 1410, Mbarara, Uganda

[6]Department of Medicine, Harvard Medical School and Massachusetts General Hospital, 125 Nashua Street, Suite 722, Boston 02114, USA

[7]Curriculum in Genetics and Microbiology, University of North Carolina at Chapel Hill, 321 South Columbia Street, Chapel Hill, NC 27516, USA

[*]Corresponding author:

Ross M. Boyce MD, MSc
Division of Infectious Diseases
University of North Carolina at Chapel Hill
130 Mason Farm Road
Chapel Hill, NC USA 27599
Phone: 919.966.2537
Fax: 919.966.6714
roboyce@med.unc.edu

Table S1: AMA primer and MID sequences

**Nested Forward Primer**	5'-GCTGAAGTAGCTGGACTCAA
**Conserved Reverse Primer**	5'-TTTCCTGCATGTCTTGAACA
**Inner Forward Primers with 5' MID**	
MID01	5'-**ACGAGTGCGT**CCATCAGGGAAATGTCCAGT
MID02	5'-**ACGCTCGACA**CCATCAGGGAAATGTCCAGT
MID03	5'-**AGACGCACTC**CCATCAGGGAAATGTCCAGT
MID04	5'-**AGCACTGTAG**CCATCAGGGAAATGTCCAGT
MID05	5'-**ATCAGACACG**CCATCAGGGAAATGTCCAGT
MID06	5'-**ATATCGCGAG**CCATCAGGGAAATGTCCAGT
MID07	5'-**CGTGTCTCTA**CCATCAGGGAAATGTCCAGT
MID08	5'-**CTCGCGTGTC**CCATCAGGGAAATGTCCAGT
MID10	5'-**TCTCTATGCG**CCATCAGGGAAATGTCCAGT
MID11	5'-**TGATACGTCT**CCATCAGGGAAATGTCCAGT
MID13	5'-**CATAGTAGTG**CCATCAGGGAAATGTCCAGT
MID14	5'-**CGAGAGATAC**CCATCAGGGAAATGTCCAGT
MID15	5'-**ATACGACGTA**CCATCAGGGAAATGTCCAGT
MID16	5'-**TCACGTACTA**CCATCAGGGAAATGTCCAGT
MID17	5'-**CGTCTAGTAC**CCATCAGGGAAATGTCCAGT
MID18	5'-**TCTACGTAGC**CCATCAGGGAAATGTCCAGT
MID19	5'-**TGTACTACTC**CCATCAGGGAAATGTCCAGT
MID20	5'-**ACGACTACAG**CCATCAGGGAAATGTCCAGT
MID21	5'-**CGTAGACTAG**CCATCAGGGAAATGTCCAGT
MID22	5'-**TACGAGTATG**CCATCAGGGAAATGTCCAGT
MID23	5'-**TACTCTCGTG**CCATCAGGGAAATGTCCAGT
MID24	5'-**TAGAGACGAG**CCATCAGGGAAATGTCCAGT

Table S2: Mean multiplicity of infection (MOI) by sub-group and ordinal logistic regression modeling of correlates of MOI

Variable	Mean MOI	OR (95% CI)	*p*-Value	aOR (95% CI)	*p*-Value
**Sex**					
Female	3.16 (2.65 – 3.67)	REF	REF	-	-
Male	3.00 (2.51 – 3.49)	0.89 (0.56 – 1.44)	0.64	-	-
**Age Category**					
<5 years	2.82 (1.89 – 3.74)	REF	REF	REF	REF
5 – 15 years	3.05 (2.56 – 3.54)	1.47 (0.73 – 2.93)	0.28	1.29 (0.51 – 3.26)	0.60
>15 years	3.26 (2.67 – 3.86)	1.64 (0.81 – 3.33)	0.17	1.28 (0.48 – 3.39)	0.63
**Parasitemia**					
<2,500/µl	3.51 (2.55 – 4.47)	REF	REF	-	-
2,500 – 9,999/µl	3.52 (2.75 – 4.29)	1.22 (0.53 – 2.84)	0.64	-	-
10,000 – 99,000/µl	2.80 (2.34 – 3.27)	0.96 (0.45 – 2.06)	0.91	-	-
≥100,000/µl	2.47 (1.79 – 3.15)	0.77 (0.32 – 1.85)	0.56	-	-
**Severity**					
Uncomplicated	3.25 (2.86 – 3.64)	REF	REF	REF	REF
Severe	2.29 (1.46 – 3.12)	0.56 (0.27 – 1.16)	0.12	0.63 (0.25 – 1.60)	0.33
**Elevation***					
Quartile 1	3.51 (2.77 – 4.26)	REF	REF	REF	REF
Quartile 2	2.70 (2.08 – 3.33)	0.51 (0.23 – 1.12)	0.09	0.43 (0.20 – 0.94)	**0.035**
Quartile 3	2.62 (2.06 – 3.19)	0.49 (0.22 – 1.08)	0.08	0.44 (0.20 – 0.98)	**0.045**
Quartile 4	1.85 (1.33 – 2.37)	0.24 (0.10 – 0.57)	**0.001**	0.23 (0.09 – 0.59)	**0.002**

Elevation ranges: Quartile 1 = 1136 – 1225m, Quartile 2 = 1259 – 1339m, Quartile 3 = 1355 – 1424m, Quartile 4 = 1451 – 1830m
Abbreviations: OR = odds ratio, aOR = adjusted odds ratio

Figure S1: Photograph showing 1 cm sections of rapid diagnostic test, circled in yellow, utilized in DNA extraction protocol.

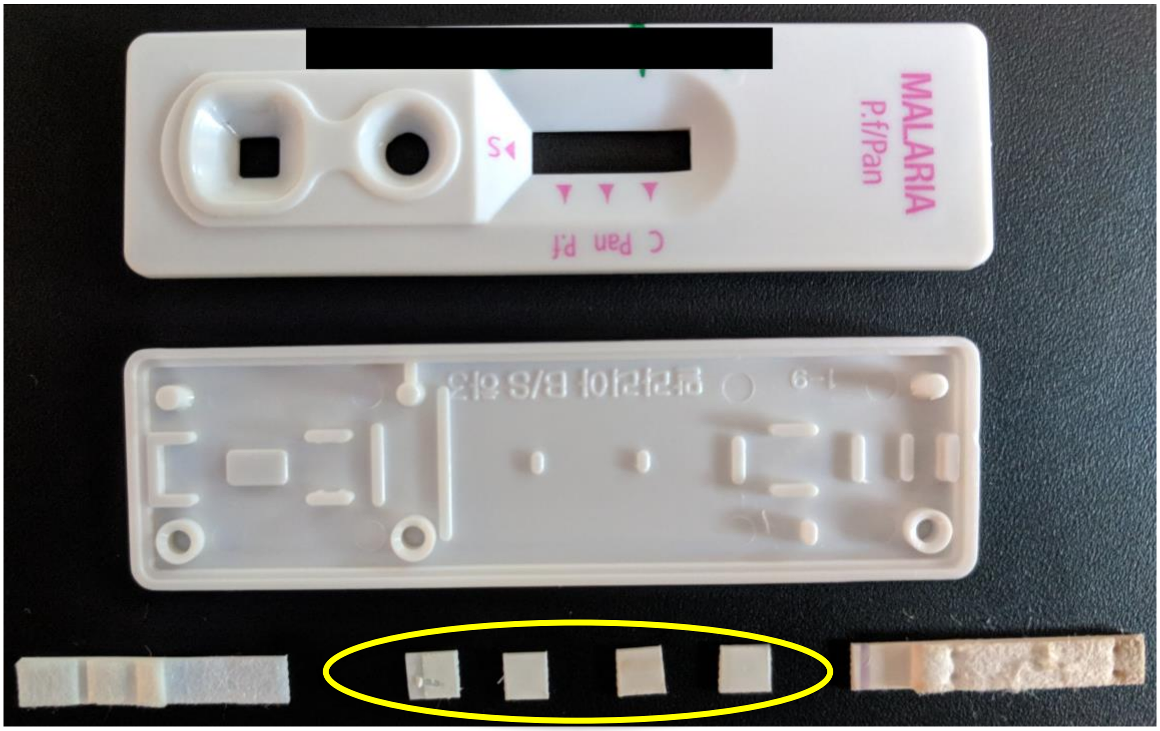

Figure S2: Summary of library construction process and final library structure

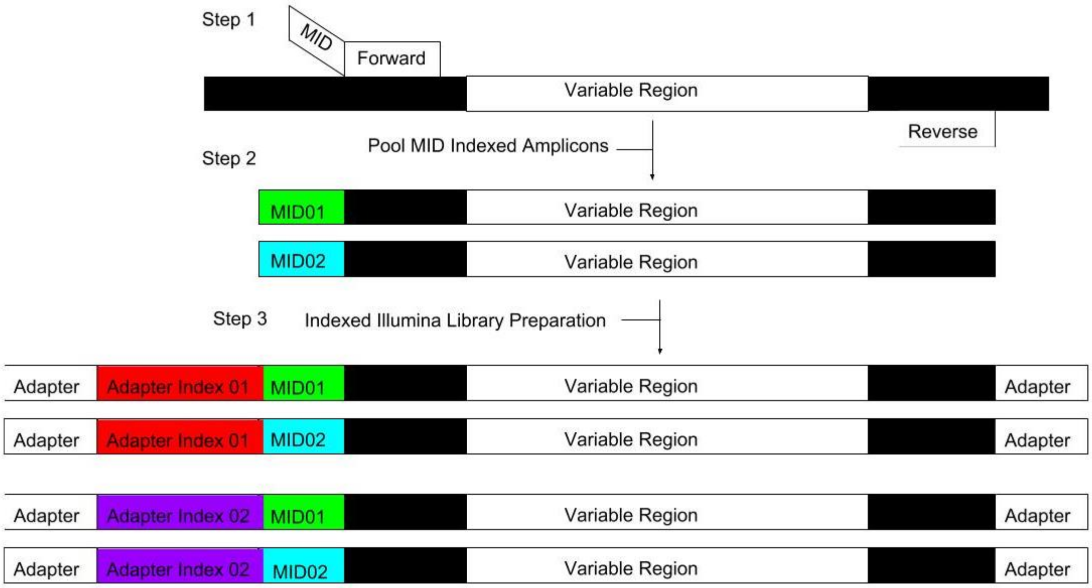

Figure S3: Allele frequencies in the five control mixtures. The number of reads per control were as follows: *Control 1* – 10,737 and 8658 reads, *Control 2* – 5,319 and 5,324 reads, *Control 3* – 6,031 and 7,705 reads, *Control 4* – 13,076 and 11,781 reads, *Control 5* – 4,331 and 7,184 reads.

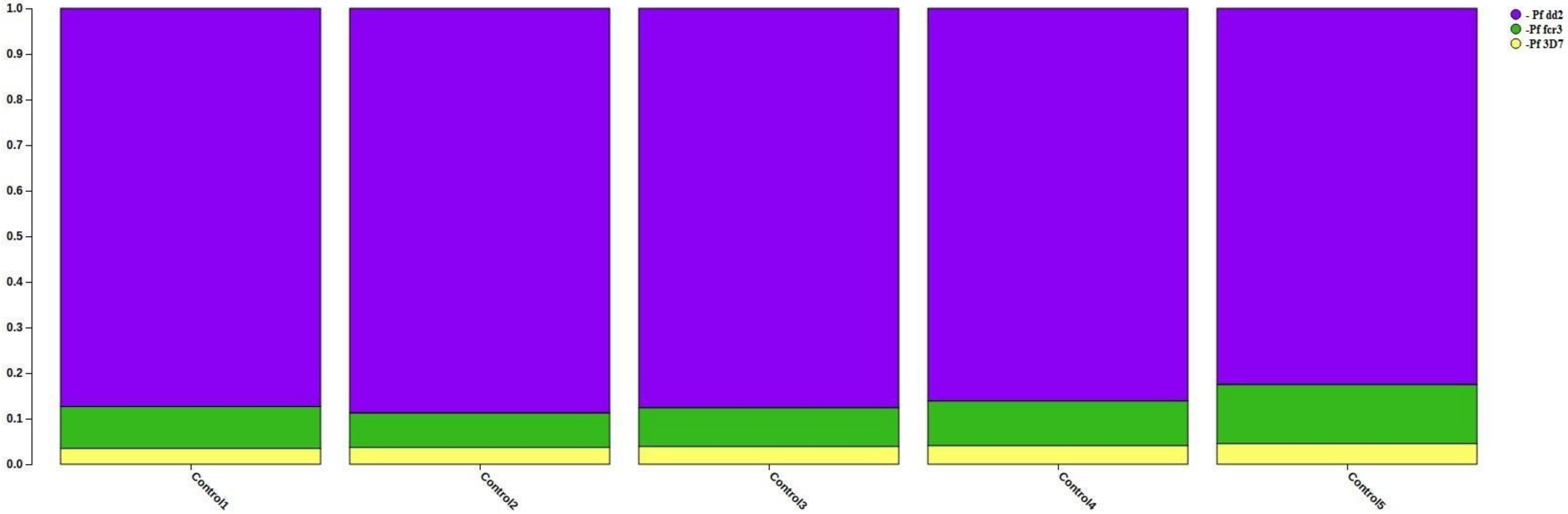

Figure S4: Estimated *ama1* allele frequencies by cloning/Sanger sequencing and deep sequencing in a three-strain (dd2, fcr3, and 3D7) control mixture. The three strains were mixed together in varying frequencies. A total of 22 colonies were Sanger sequenced. For deep sequencing, *ama1* amplicons were generated in quintuplicate and a total of 80,971 reads passed quality filtering and were used for haplotype determination. Estimated allele frequencies by cloning and Sanger sequencing were dd2=0.64, fcr3=0.27 and 3d7=0.09. Estimated allele frequencies by deep sequencing were dd2=0.85, fcr3=0.10 and 3d7=0.04. The bootstrap method with 1000 replicate bootstraps was used to estimate 25% and 97.5% confidence intervals for both sequencing results.

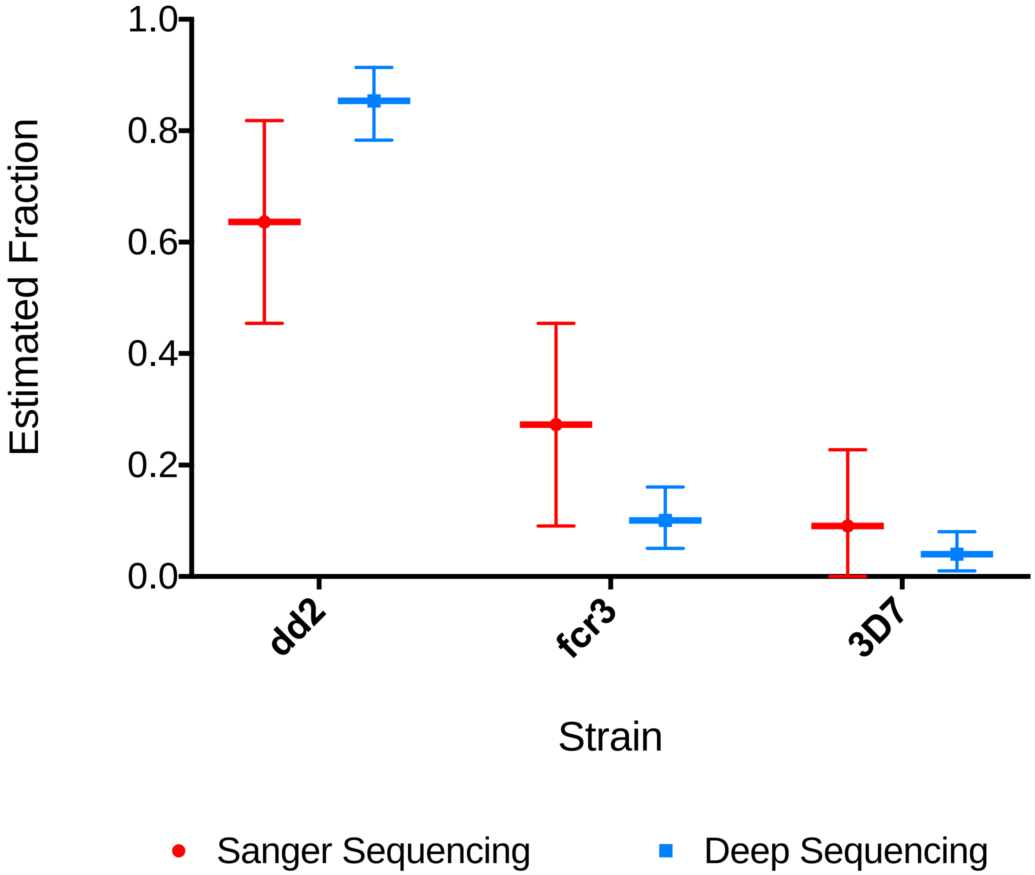

Figure S5: Total read depth of each sample stratified by multiplicity of infection (MOI)

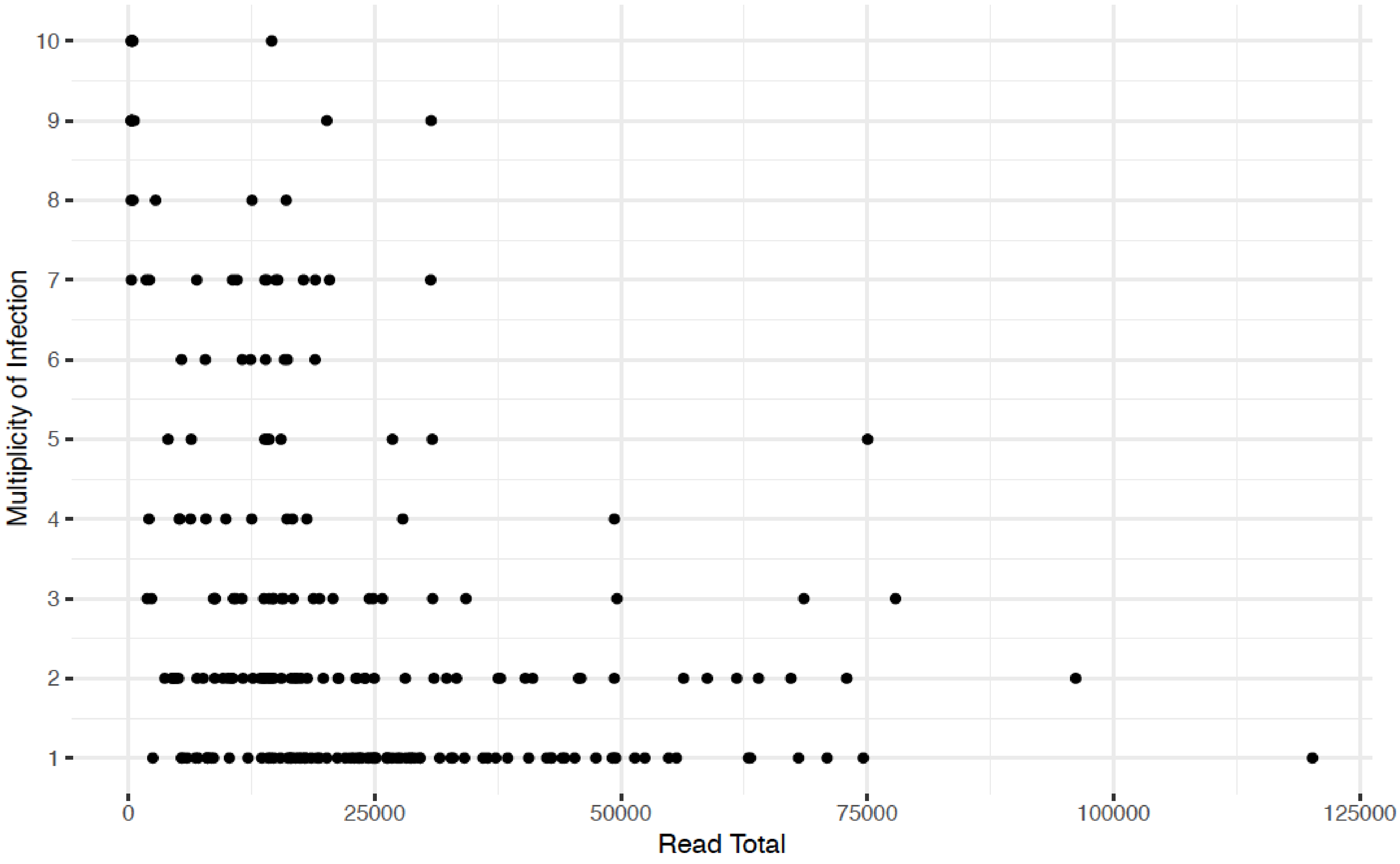

Figure S6: Histogram showing the absolute number of samples in which each haplotype (n=39) was found.

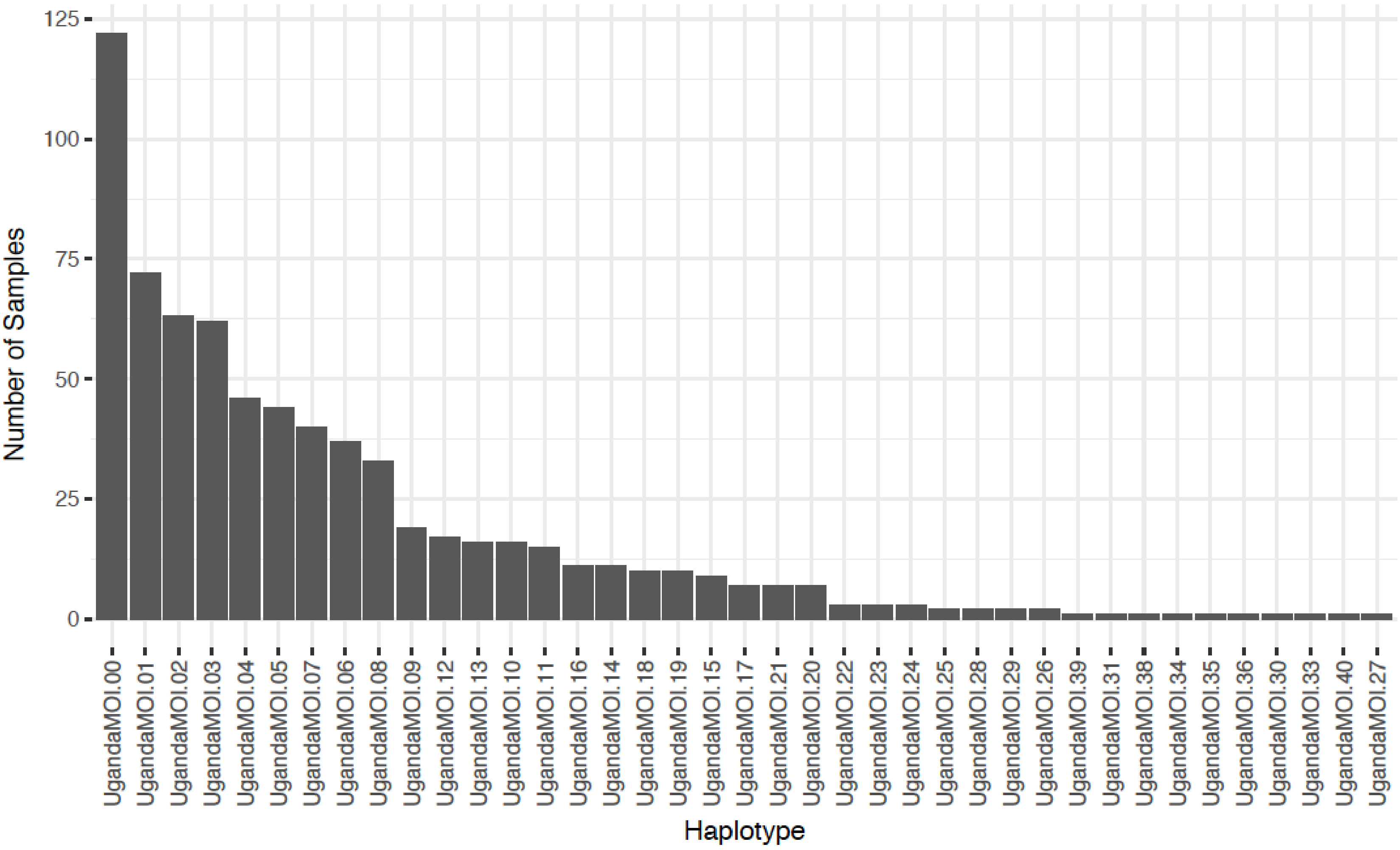

Supplement: Supplementary file 1 — Supplementary Information [file 41598_2018_28534_MOESM1_ESM.pdf]
